# Prenatal features of aneurysm of the posterior cerebral artery: a case report

**DOI:** 10.1186/s12884-023-06056-9

**Published:** 2023-10-17

**Authors:** Pakorn Chaksuwat, Phudit Jatavan, Srimeunwai Ake-sittipaisarn, Nonthawat Chetprayuk, Theera Tongsong

**Affiliations:** 1 Department of Obstetrics and Gynecology, Sawanpracharak Hospital, Nakhon Sawan, Thailand; 2 Department of Obstetrics and Gynecology, Faculty of Medicine, Chiang Mai University, Chiang Mai 50200, Chiang Mai, Thailand

**Keywords:** Aneurysm, Case report, Posterior cerebral artery, Pregnancy, Prenatal diagnosis, Ultrasound

## Abstract

**Background:**

Fetal cerebral aneurysm other than aneurysm of vein of Galen aneurysmal malformation (VGAM) is extremely rare. This report describes prenatal features of aneurysm of the posterior cerebral artery (APCA) with rapid progression and its natural intrauterine course of the disease, which has never been reported.

**Case presentation:**

This is the first report of prenatal features of APCA, detected at 34–36 weeks of gestation, simulating choroid plexus cyst or arachnoid cyst. The diagnosis was based on color flow ultrasound with tracing along the course of cerebral arteries. Also, rendered 3D color flow ultrasound was helpful in demonstrating course of the vessels feeding the aneurysm and supporting the diagnosis. The aneurysm showed nature of rapidly progressive changes, leading to leakage resulting in intracerebral and intraventricular hemorrhage as well as high output state associated with anemia. Prenatal diagnosis and management are very challenging. This case ended up with planned delivery at 37 weeks, giving birth to a surviving male newborn, weighing 2600 g. The neonatal CT brain scans and CTA confirmed the prenatal findings. The prognosis was relatively poor because of extensive intracerebral hemorrhage with severe hydrocephalus and brain midline shift. The couple opted for neonatal palliative care without neurosurgical correction.

**Conclusion:**

This study demonstrate that the most important tool for prenatal diagnosis is color Doppler ultrasound, which will demonstrate turbulent blood flow. Three-dimension color Doppler ultrasound is helpful in supporting the diagnosis. The case presented here suggests that the disease has a natural course of rapid progression and massive brain destruction or high output congestive heart failure can be expected.

**Supplementary Information:**

The online version contains supplementary material available at 10.1186/s12884-023-06056-9.

## Introduction

Fetal cerebral aneurysm is a rare but very challenging. Nearly all cases of prenatal diagnosis of cerebral aneurysm ever reported were vein of Galen aneurysmal malformation (VGAM), with the estimated incidence of 1 in 10,000 to 1 in 25,000 births [1]. Fetal intracranial aneurysms of other vessels are extremely rare. To the best of our knowledge, prenatal diagnosis of aneurysm of the posterior cerebral artery (APCA) and its natural intrauterine course of the disease has never been reported, whereas the fetal-type posterior cerebral artery abnormalities or aneurysm is a common neurovascular variant in adult [2–4]. Though never been studied, APCA can theoretically cause fetal heart failure as manifested by hydrops with a high mortality rate as described in VGAM [5], whereas intracranial hemorrhage is a rare finding in VGAM [6]. This report describe prenatal features of APCA with rapid progression leading to aneurysmal leakage and brain destruction as well as high output state.

## Case presentation

**Fig. 1:**
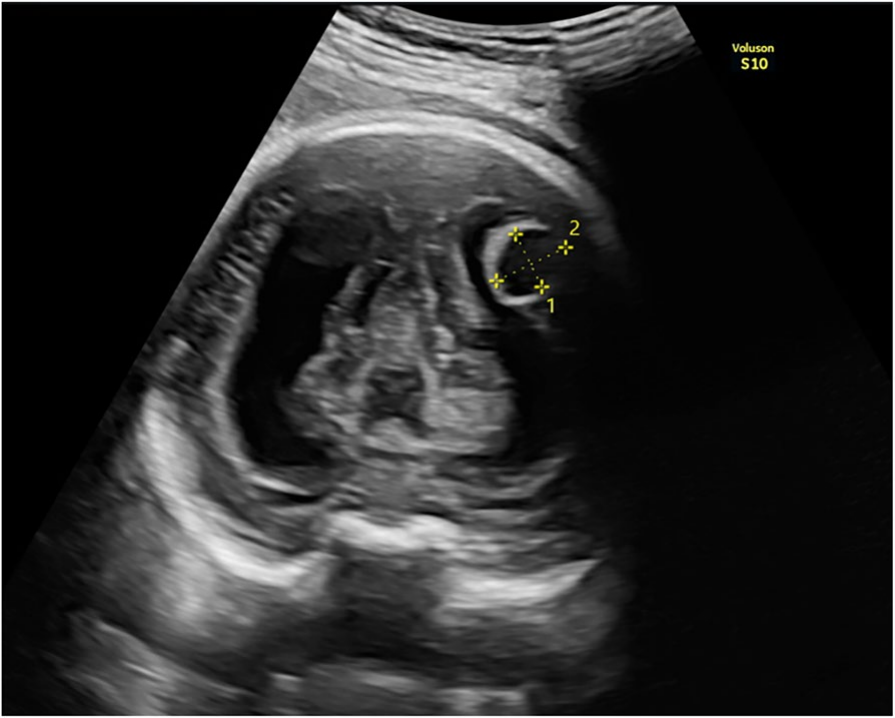
Oblique coronal scan of the posterior fetal head at 34 weeks of gestation shows well-circumscribed anechoic cyst 1 cm diameter, protruding from the left lateral wall of the lateral ventricle

**Fig. 2:**
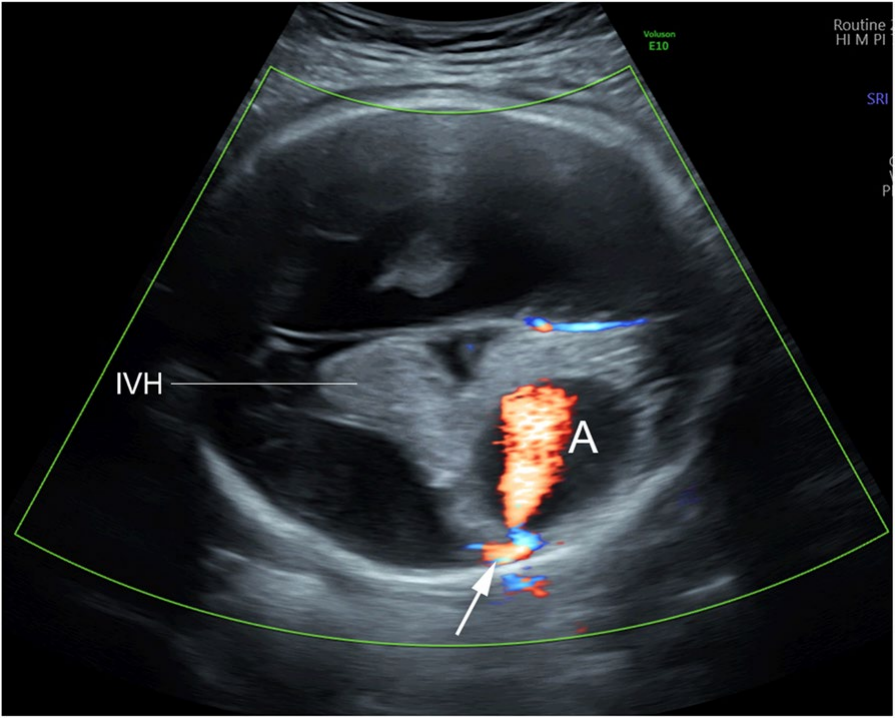
Color flow mapping at transventricular view at 36 weeks of gestation shows cystic mass with progressive enlargement and active flow jet originating from lateral wall of the lateral ventricle (arrow) with pericystic and intraventricular hyperechoic area representing hemorrhage. (A: aneurysm; IVH: intraventricular hemorrhage)

**Fig. 3:**
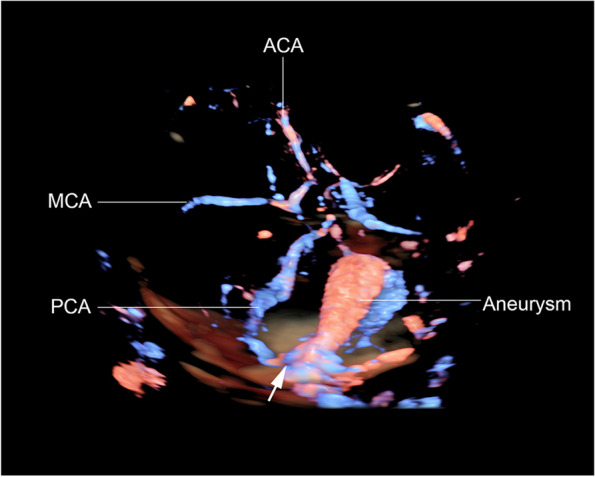
Rendered 3D-color flow ultrasound of the fetal brain at 36 weeks of gestation shows course of posterior cerebral artery (PCA) feeding the aneurysm from lateral wall of parieto-temporal hemisphere (arrow: indicating originating point of flow jet feeding the aneurysm). (ACA: anterior cerebral artery; MCA: middle cerebral artery)

**Fig. 4:**
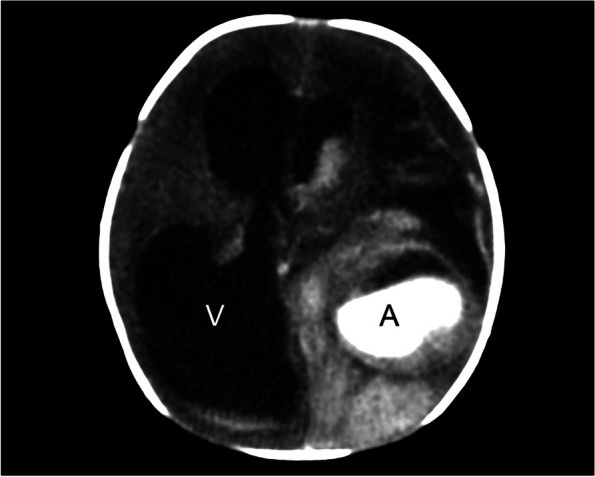
Neonatal CT brain with contrast on day 1 after birth shows marked ventriculomegaly (V) and bright hyperechoic area representing active flow in the aneurysm (A), surrounded by hyperechoic area of hemorrhage

A 28-year-old primigravid was referred for detailed fetal ultrasound at 36 weeks of gestation due to suspicion of fetal ventriculomegaly. Her obstetric and medical history as well as familial history were unremarkable. She had not been exposed to any known teratogens and had no clinical sign of TORCH infection. Her marriage was not consanguineous. The current pregnancy course was uneventful. Mid-pregnancy anomaly ultrasound screening was unremarkable. Ultrasound at the referring hospital at 34 weeks of gestation showed a male fetus with dilated bilateral lateral ventricles (18 mm, and 10 mm width), third ventricle and fourth ventricle, and a round shape, anechoic cyst (13 × 10 mm), at the left parieto-occipital lobe adjacent to the left lateral ventricle (Fig. 1). However, the color Doppler ultrasound was not applied. All structures were otherwise normal. Fetal biometry revealed appropriate for gestational age. Serologic tests for TORCH infection were negative. On follow-up ultrasound at 36^+5^ weeks at our hospital, the size of lateral ventricles, third ventricle and fourth ventricle were relatively the same. The round -shape anechoic cyst at the left parieto-occipital lobe was increased in size, 32 × 27 × 25 mm, with expanding hyperechoic area surrounding the cyst, representing pericystic hemorrhage. The left lateral ventricle was filled with hyperechoic irregular mass (hemorrhage) in most part. Color and spectral Doppler ultrasound showed turbulent flow in the cyst. The originating point of arterial jet arose from the left posterior cerebral arteries at left lateral border of temporal-parietal area of the hemisphere (Fig. 2). With color Doppler flow, the course of the left posterior cerebral artery could be traced from the circle of Willis to the aneurysm. Three-dimension color flow ultrasound was helpful in confirming the diagnosis (Fig. 3). The main prenatal diagnosis was aneurysm the left posterior cerebral artery with leakage leading to periventricular and intraventricular hemorrhage and pressure effect on the hemisphere. Fetal cardiomegaly and hepatomegaly (cardiac diameter as well as circumference and liver length were of greater than 95^th^ percentile of reference ranges) were also documented. Peak systolic velocity of the middle cerebral artery was 103.5 cm/s (greater than 1.5 MoM). After multidisciplinary consensus (neonatologists, pediatric neurosurgeons, and maternal–fetal medicine team) and patient counseling about poor prognosis in case of no intervention, planned delivery for postnatal management was performed by induction of labor at 37 weeks of gestation. However, cesarean section was performed due to failure of induction, giving birth to a male newborns, weighing 2,600 gm with Apgar scores of 9, 10 and 10 at 1, 5 and 10 min, respectively. Grossly, the newborn was not obviously abnormal except for pale skin. The occipitofrontal circumference was 37 cm. The fetal movement was normal. Neonatal CT brain scans on day 1 (Fig. 4) and CTA of the neonatal brain on day 5 after birth showed as follows: The arteriovenous malformation in left parietal lobe, fed by left middle and left posterior cerebral arteries, drained into cortical vein and left transverse sigmoid sinuses, with intervening arteriovenous malformation nidus, 12 × 13 × 12 cm in size. Intranidal saccular aneurysm sized 48 × 55 × 50 mm. A heterogeneous hyperdense lesion of intra-perenchymal hematoma at left perieto-tempero-occipital lobes. Encephalomalacia at left fronto-parieto-temperal lobes. Diffuse brain atrophy. Moderate amount of intraventricular hemorrhage in left lateral ventricle and third ventricle. Severe hydrocephalus with rightward bowing of the midline structures with no brain herniation was noted. The complete blood count showed fetal anemia; hematocrit and hemoglobin level of 32.5% and 10.3 g/dl respectively, and normal platelet concentration. The neonatal chest film x-ray, including abdomen, showed mild cardiomegaly and hepatomegaly. Though the baby survived and well received breast feeding, the prognosis was relatively poor. The couple opted to have palliative and supportive care at home, without neurosurgical correction. The baby developed severe hydrocephalus with gradually worsening and died on 46^th^ days of life.

## Discussion

This case report describe new insights, as follows: 1) Because of no anomaly seen at mid-pregnancy screening and first detection as a small cyst at 34 weeks, it suggests that APCA likely occur in the third trimester, as commonly seen in VGAM [1, 5]. 2) APCA tends to have rapid progression, change from a small simple cystic mass to enlarged cyst with turbulent flow and leakage, leading to extensive brain destruction. 3) Spontaneous bleeding in utero with massive hemorrhage, resulting in anemia (MCA 2 MoM). Unpredictable rupture could occur any time. Surveillance of the mass is required for early intervention if needed. APCA should be considered as a serious lesion of the fetal brain with a nature of rapid progressive change and vulnerability of leakage or rupture. 4) Different locations of fetal brain aneurysm might have different natural course of progression, the risk of rupture/leakage and prognosis. Therefore, tracing course of the feeding arteries is an essential part of assessment. Certainly, color Doppler ultrasound is the must-have examination and 3D color flow Doppler is likely helpful, as presented in this case. 5) In addition to rupture, heart failure, which is the main factor of poor prognosis in VGAM [7], might be expected in APCA, as evident by cardiomegaly and hepatomegaly. However, notably, leakage / rupture of APCA, which is rare in VGAM [6], preceded heart failure. Therefore, this case signifies that APCA may possibly be more vulnerable to rupture than VGAM. 6) Not only does the aneurysm destroy brain parenchyma through compression and hematoma, but they also can result in severe anemia, high-output heart failure and create areas of ischemic insult through the steal phenomenon as indicated by severe hydrocephalus of the contralateral hemisphere.

The differential diagnosis of a fetal intracranial vascular mass includes hemangioma, Kaposiform hemangioendothelioma, angioblastoma, angiosarcoma, congenital hemangiopericytoma, and arteriovenous malformation / aneurysm [8]. Doppler analysis demonstrating blood flow within the lesion is key to differentiating an arteriovenous malformation / aneurysm from other cystic lesions [1].

Natural course of progression of the aneurysm in utero has never been studied. Nevertheless, our evidence supports the progressive nature as described in postnatal life. In adults, most cerebral aneurysms develop rapidly in hours, days or weeks, to achieve a size, of which elasticity in the aneurysmal wall can no longer expand, leading to either rupture or undergoing stabilization and hardening [9–11]. This is because the non-ruptured aneurysms can gain more tensile strength secondary to compensatory formation of excessive collagen. Accordingly, the tendency of rupture decreases since then, unless the aneurysmal size is relatively enlarged at the beginning of hardening or stabilization. Typically, aneurysms with size of greater than 1 cm in diameter at the beginning of stabilization take higher risk of subsequent growth and rupture since wall tension increases with the square of the diameter, according to Laplace's law. Accordingly, rapidly progressive change with relatively large size in our case might be predictive of rupture. Additionally, we hypothesize that thinned-wall of fetal cerebral arteries together with high velocity of arterial flow because of low viscosity secondary to fetal anemia and compensatory high-output state caused by the aneurysm might facilitate the rupture.

In summary, fetal aneurysm of the APCA is extremely rare, possibly occurring in the third trimester of pregnancy as commonly seen in aneurysm of the vein of Galen. This disorder must be differentiated from other cystic brain anomalies. The most important tool for prenatal diagnosis is color Doppler ultrasound, which will demonstrate turbulent blood flow. Three-dimension color Doppler ultrasound is helpful in supporting the diagnosis. The case presented here suggests that the disease has a natural course of rapid progression and massive brain destruction or high output congestive heart failure can be expected. However, more several case reports/series are warranted to establish the true natural intrauterine course and prognosis.

### Supplementary Information

**Additional file 1.**

## Data Availability

The data of this report are available from the corresponding authors upon request.
